# Integrating spatial technology into studying the generational differences of migrants’ health protection status in urban China

**DOI:** 10.1186/s12939-015-0159-x

**Published:** 2015-03-10

**Authors:** Yang Yang, Xing Zhao, PeiYuan Qiu, Xiao Ma, Chih-Ping Chou

**Affiliations:** West China School of Public Health, Sichuan University, No. 17, Section 3, South Renmin Road, Chengdu, Sichuan 610041 China; Department of Preventive Medicine, Keck School of Medicine, University of Southern California, Los Angeles, CA USA

## Abstract

**Objectives:**

The objectives of this study were to investigate differences on health protection status between two generations (born pre- vs. post- 1980) of rural-to-urban migrants in China, and whether the differences are associated with spatial contexts.

**Methods:**

Respondent-Driven Sampling (RDS) approach was used to recruit migrants in Chengdu city from September 2008 to July 2009. All migrants’ residences were geo-coded on the map. Hepatitis B Vaccination serves as a surrogate for the Health protection status. Logistic regression was used to explore the association between independent variables and the Hepatitis B vaccination status. Spatial scan statistics were used to explore the spatial pattern of the Hepatitis B vaccination status.

**Results:**

Among the 1045 rural-to-urban migrants, higher education, better employment condition and post-80 generation are positively associated with the Hepatitis B vaccination status, while marriage status, the insurance status and the income are not. The spatial scan statistics identified three spatial clusters of low vaccination rate. Two of them were in urban villages and the other was a declining workers’ community.

**Conclusions:**

The migrant population is heterogeneous, and the post-80 generation migrants get more health protection. Spatial analytical techniques illustrated clusters of low vaccination rate are highly linked with pre-1980 generation migrants and other socioeconomic factors, especially the employment condition. Such information might shed light on the differences and needs across migrant subgroups and may be useful for developing more targeted health policies for Chinese migrants.

## Introduction

The scale of world migration has expanded remarkably over the past decades. As a result, population mobility becomes one of the leading global policy issues for the 21st century [1]. Since the economic reforms of China in 1978, a growing number of rural populations are working in urban area [2]. The volume of rural-to-urban migrants has increased dramatically in China with the rapid economic growth for the past 30 years, and it amounts to around 260 million migrants according to the 2010 national census.

The continuing trend of rural to urban migration leads great attentions to health issues of the migrants in research with most existing studies focused on the risks of diseases and the access to health service. For the risks of diseases, current studies concentrate on infectious diseases, such as Human immunodeficiency virus (HIV) and Tuberculosis (TB). Several studies found that migrants lack the knowledge of HIV and are more likely to be engaged in risk behaviors, such as casual sex without using condoms [3,4]. Migrants also have higher prevalence of TB than local residents [5,6]. In addition, migrants with TB are usually delayed for treatment and have lower cure rates [6,7]. Occupational diseases and maternal health are also related to migrants [8,9]. For the access to the health services, studies found that migrants had limited access to regular medical services, as urban health services are usually not available for rural-to-urban migrants in China. In 2011, only 16.7% of migrant workers in China have urban health insurance [10]. Because of the lack of insurance coverage, migrants face high cost of health care and unsupervised self-treatment or substandard care [11,12]. Furthermore, many migrant women did not receive adequate antenatal care, with the antenatal care initiated later than the optimal first 12 weeks of pregnancy [13].

One limitation of existing studies is viewing migrants as a homogeneous group, and seldom examining the differences and needs across migrant subgroups, such as the pre- and post-1980 generations. The young, or post-1980, generation born after 1980 has been increasingly discussed by the government and academic communities in China. Compared with the old, or pre-1980, generation, the post-80 generation is more individualistic and westernized with better education, higher income, and greater awareness of rights and social equality [14]. Hence, the young generation migrants have higher socioeconomic status than the old generation. Although higher socioeconomic status is usually associated with better health [15], few studies have combined both socioeconomic influences and generational impact on health disparities in China which is the main focus of this article.

Another motivation of this study is from the “segmented assimilation” theory, originally proposed by Portes and Zhou [16]. In 2000s, researchers examined the spatial dimension of “segmented assimilation” theory and found that immigrants’ spatial dimension are closely related to their social status as well as their health status [17-19]. The spatial segmented assimilation suggests the importance of urban space on the SES difference of migrant subgroups. To better understand the heterogeneity among migrants group in China, we incorporate the spatial dimension in the analysis.

In our work, Hepatitis B Vaccination (HBV) serves as a surrogate for the Health protection status. The ‘China National Hepatitis Epidemiological Survey 1992’ found that the prevalence of HBsAg for population aged 1–59 was 9.8%, or about 120 million people were carrying HBsAg [20]. Due to the high prevalence of Hepatitis B, the immunization was first recommended for routine vaccination of infants in China in 1992. However, this policy was primarily implemented in wealthier eastern provinces until 2002 [21]. Our participants were not covered by newborn infants Hepatitis B Immunization Program, as they are from western rural areas, and 99% of them were born before 1992.

In summary, this study aimed to investigate whether there are differences between young and old migrant generations on health protection status in the urban setting, and whether the differences are associated with spatial contexts.

### Data and measure

Data used in this study were from the “Applying RDS sampling to research migrant population’s social network and their needs for health services” project supported by Chinese National Science Foundation. The data were collected from September 2008 to July 2009 in Chengdu, Sichuan Province, China. Chengdu is the center of economy, transportation and communication in western China. The urban area has more than 14 million inhabitants: more than half live within the metropolitan area while others in the surrounding region. From the 2010 census, there were 2,620,640 migrants in the city. Respondent-Driven Sampling (RDS) approach was used in an effort to recruit a representative sample of migrants. RDS is a chain-referral procedure. A total of 12 seeds were selected after considering gender, age, occupation, and living sites. Each seed was given three coded coupons to recruit peers. We then consented and enrolled persons who presented one of these valid coupons and who we deemed eligible; in turn, each new enrollee was given three coded coupons for the purpose of recruiting peers. The method is superior to other snowball sampling techniques by incorporating sampling procedures and analytical tools that allow for calculating unbiased population estimates [22], and some relevant methodological assessment for our data had been made regarding the geographically representation [23]. A total of 12 seeds were selected after taking gender, age, occupation, and living sites into consideration. The whole process was a chain-re-referral procedure. In total, 1055 rural-to-urban migrants were recruited. Among them, 10 people reported being infected by Hepatitis B, and had been excluded for the analysis. The final sample of this study consisted of 1045 rural to urban migrants.

Assessments were conducted through face-to-face interview during participant’s visit to the research site. The dependent variable is whether the participants have Hepatitis B vaccination ever before (0: No; 1: Yes). In addition to the Hepatitis B vaccination status, social demographic data, such as age, gender, education, employment condition, were obtained. The key independent variable is the dichotomous measure of generation. The participants born after January 1, 1980 were grouped into the young generation migrants, while the others were in the old generation. The Socioeconomic status (SES) variables contain income, educational attainment and employment condition. The income is measured by personal monthly income (in the unit of ¥1000). We categorized education into two groups: middle school or below (<=9 years) and high school or above (>9 years). The employment condition was divided into three groups [24]: Formal employment, informal employment, and precarious employment. Formal employment indicates employment with labor contract. Migrants without any labor contract but with fixed monthly income were grouped into informal employment condition; while those without labor contract and fixed monthly income were grouped into precarious employment condition.

Moreover, the current residential address for each migrant was recorded. In order to visualize the spatial pattern of migrants, we selected a 2008 version government-authorized Chengdu metropolitan area map. With the guidance from the relevant expert, we defined the region within the first and second ring roads as the downtown area; region between the second and the third ring roads as the suburban area; and region outside the third ring road as the exurban area. Main roads and boundaries were used to divide central areas of Chengdu into 26 areas (Table 1). The area coding together with migrants’ current residential address was used to visualize the geographical distribution of migrants. The residential information of three migrants was missing and therefore, only 1042 individuals were geocoded, instead of 1045.

**Table 1:** **GIS codes of the map**

	**Area**	**GIS code**
Downtown	With the first ring road	1,2,3,4
	Between the first and the second ring roads	5,6,7,8
Suburban	Between the second and the third ring roads	9, 10, 11, 12
Exurban	Out of the third ring road	13-26

### Ethics statement

The study was approved by the Ethics Committee of No.4 West China Teaching Hospital (West China School of Public health), Sichuan University. Written informed consent was obtained from all the study participants, and all the data analyzed were anonymized for the confidentiality.

### Statistical methods

Two statistical methods were used in this study to investigate Hepatitis B vaccination status: Descriptive analysis was conducted to examine the frequency distribution and the odds ratio for the categorical variables, or mean values and standard deviations for the continuous variables. Logistic regression was performed to explore the association between Hepatitis B vaccination and independent variables: generation, insurance status, gender, education, marriage, employment condition and income. In the process of logistic analysis, univariate analyses were conducted first to screen independent variables which were associated with Hepatitis B vaccination status with a *P* value less than 0.2 to be included as covariates in the multivariate logistic regression model. And finally the spatial scan statistics were used to explore the spatial pattern of the Hepatitis B vaccination status. Data analyses were performed using SAS.

With the geo-coding of migrants on the map, the spatial scan statistic [25,26] was used to identify areas of lowered rate of vaccination using the likelihood ratio test. The hypothesis is that the unvaccinated migrants were distributed randomly over space. The spatial scan technique imposes a circular form on the clusters, but allows them to be centered in any of the different individual locations and take any possible size (with a maximum size of 50% of all individuals). This approach tests the statistical significance of potential clusters with a likelihood ratio statistic, whose distribution under the null hypothesis is obtained through the Monte Carlo simulation. This strategy allows one to derive a *P* value for each potential cluster. Because of the case-control data structure, the Bernoulli model based spatial scan statistic is employed. Finally, a contrast of the characteristic covariates between the detected clusters of lowered rate of vaccination and the remaining area is presented. The spatial analysis the SatScan 9.11, while and the final visualization of spatial clusters was created utilizing the ArcGIS.

## Results

### Descriptive statistics

The descriptive statistics of univariate analyses are presented in Table 2. The new generation consists of 53.5% (559/1045) of the study sample. Formal employment and informal employment account for 16.2% (169/1045) and 34.9% (365/1045), respectively. While the difference in rates of vaccination between men and women is small, other covariates showed substantial difference. Having high education, being unmarried status and being in the Post-1980 generation are more than double or even triple the proportion of vaccination compared to their corresponding reference groups. Their odds ratio estimates range from 2.98 to 3.5. Furthermore, compared to the group of precarious employment, the informal employment increases the vaccination rate from 23.53% to 35.74%, with another 12% increase (47.84%) for the formal employment group. Having insurance also promotes the vaccination rate by approximate 14%.

**Table 2:** **Univariate descriptive statistics**

	**N = 1045**	**Unvaccinated (N = 651)**		**Vaccinated (N = 394)**		**Odds ratio or mean difference**	**95% confidence interval**	
**Variable**		**No. or mean**	**% or (standard deviation)**	**No. or mean**	**% or (standard deviation)**			
Generation	Pre-1980 generation	419	74.96	140	25.04	--		
	Post-1980 generation	232	47.74	254	52.26	3.277	2.523	4.255
Gender	female	348	63.74%	198	36.26%	--		
	male	303	60.72%	196	39.28%	1.137	0.885	1.46
Education	middle school or below	503	72.37	192	27.63	--		
	high school or above	148	42.29	202	57.71	3.576	2.731	4.682
Marriage	others	474	71.71	187	28.29	--		
	unmarried	177	46.09	207	53.91	2.964	2.28	3.853
Employment condition	precarious employment	117	76.47	36	23.53	--		
	informal employment	365	64.26	203	35.74	1.808	1.198	2.727
	formal employment with labor contract	169	52.16	155	47.84	2.981	1.934	4.594
Insurance status	No	523	65.79	272	34.21	--		
	Yes	123	51.9	114	48.1	1.782	1.328	2.391
Income		1.17	(1.13)	1.42	(1.48)	0.252	0.09	0.41

--. Reference group.

### Logistic regression

In our analysis, only gender was screened out in the univariate analysis, and the results of the multivariable logistic regression analysis were summarized in Table 3. Marriage status, the insurance status and the income were not considered to have influence on vaccination status as their 95% confidence interval of the odds ratio contain 1. Those having formal employment with labor contract or having informal employment were likely to take vaccination, with odds ratio 1.92 (C.I. = 1.14, 3.22) and 1.82 (C.I. = 1.16, 2.84), respectively, compared to the precarious employment counterpart. Moreover, the Post-1980 generation still increased the likelihood for vaccination after taking account the socioeconomic information, with odds ratio 1.98 (C.I. = 1.33, 2.95).

**Table 3:** **Odds ratios of the logistic regression model**

**Parameter**		**DF**	**Estimate**	**Standard error**	**Wald**	**Pr > ChiSq**	**Odds ratio**	**95% confidence interval**	
Intercept		1	-1.87	0.22	71.43	<.01			
Education	high school or above	1	0.67	0.17	14.82	0.01	1.96	1.39	2.76
Marriage status	unmarried	1	0.20	0.21	0.95	0.33	1.22	0.82	1.83
Income		1	0.09	0.05	2.78	0.10	1.09	0.98	1.22
Employment condition	precarious employment			--					
	informal employment	1	0.60	0.23	6.85	0.01	1.82	1.16	2.84
	formal employment with labor contract	1	0.65	0.26	6.10	0.01	1.92	1.14	3.22
Generation	new generation	1	0.68	0.20	11.15	0.01	1.98	1.33	2.95
Insurance status	Yes	1	0.16	0.20	0.61	0.43	1.17	0.79	1.73

--. Reference group.

### Spatial cluster analysis

**Figure 1:**
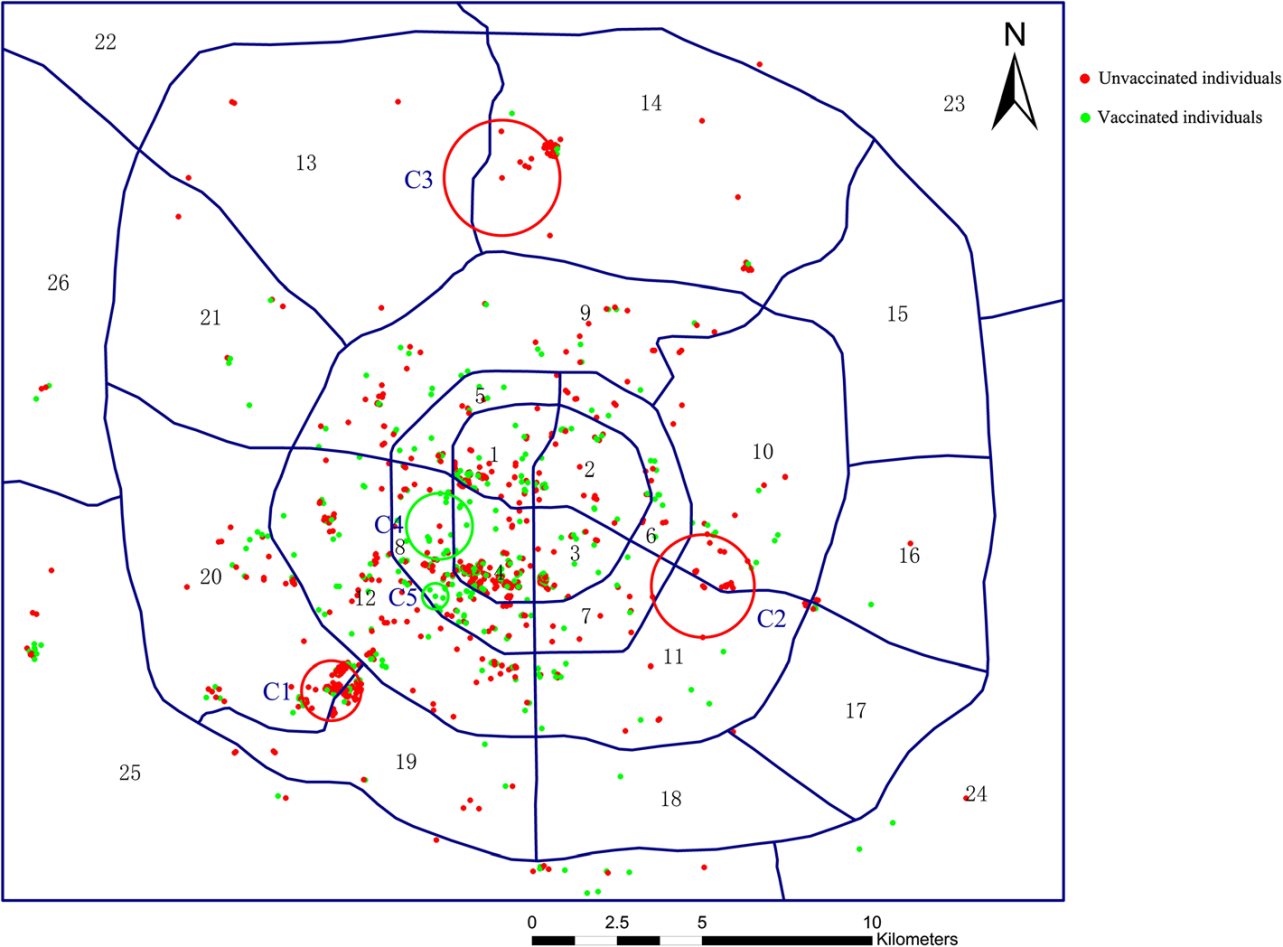
**Spatial clusters of low or high vaccination rate.**

The spatial scan statistics identified three spatial clusters of low vaccination rate and two clusters of high vaccination rate (see red circles and green circles, respectively, in Figure 1), and the corresponding statistics were presented in Table 4. With the significance level of 0.05, only one cluster of low vaccination rate was detected. The cluster is concentrated at the intersection between districts 19 and 20 and is located outside the third ring road of the city in the exurban area. This cluster belongs to an urban village. Besides, there are two other clusters of low vaccination rate with the *P* value of 0.077. They are both made up of 19 individuals without vaccination, and have the relative risk of 1.62. One is in district 14 and the other is at the intersection of districts 10 and 11. The cluster in district 14 is also outside the third ring road and also is an urban village. The cluster in districts 10 and 11 are between second and third ring road, and it is mainly a declining workers’ community. The two clusters of high vaccination rate are interesting, although their *P* value is not small. They are both in district 8, inside the second ring road (downtown area).

**Table 4:** **Summary information of clusters**

**Cluster**	**Population**	**Number of cases**	**Expected cases**	**Observed/expected**	**Relative risk**	**Log likelihood ratio**	***P*** **value**
1	94	80	58.73	1.36	1.41	12.80	0.0023
2	19	19	11.87	1.60	1.62	9.04	0.077
3	19	19	11.87	1.60	1.62	9.04	0.077
4	21	4	13.12	0.30	0.30	8.49	0.152
5	7	0	4.37	0	0	6.90	0.553

We define the three red clusters as the regions of low vaccination rate. To contrast the regions of low vaccination rate with the remaining regions, we summarized the characteristics of the four significant covariates in the last multivariate logistic regression (Table 5). The result are consistent with that of the preceding regression analysis in the sense that: the regions with low vaccination rate versus the remaining regions have a higher proportion of migrants with middle school or lower education level, precarious employment and informal employment, and born before 1980.

**Table 5:** **Contrast of characteristic variables between clusters and the remaining area**

		**Low vaccination clusters (%)**	**Other regions (%)**
Education	high school or above	10 (7.6%)	339 (37.3%)
	middle school or below	122 (92.4%)	571 (64.7%)
Employment condition	precarious employment	35 (26.5%)	117 (12.9%)
	informal employment	79 (59.8%)	486 (53.4%)
	formal employment with labor contract	18 (13.7%)	307 (33.7%)
Generation	Post-1980 generation	21 (15.9%)	462 (50.8%)
	Pre-1980 generation	111 (74.1%)	448 (49.2%)

## Discussion

Our work demonstrates the positive associations between the Hepatitis B vaccination and educational level and employment condition. This is consistent with previous studies in the sense that greater educational attainment contributes to better health [27,28]. It is plausible for the employment condition, as the migrants with labor contract have better health protection than other migrants, meaning employment condition is a social determinant of health [24] for migrants in China. Meanwhile, the generation variable (born before vs. after 1980) is still associated with the vaccination status after adjusting for the socioeconomic variables. Future research could be conducted to explore variables are associated with generation, such as attitudes towards preventive measures. Marriage status, insure status and income were not found to be significantly associated with vaccination status in this study. These findings were not surprising, as there are strong associations between these three variables and generation, education and employment status. First, the unmarried migrants are primarily born after 1980 with better education. Second, the insure status is positively associated with the education and the employment condition. The study does not get a statistically significant result for the income. There may be two reasons. First, the income is highly associated with the other variables, such as education, employment condition. Usually the job with the contract tends to have a better salary, while it is trivial that the higher education probably leads to better jobs. Second, the income data may not be particularly accurate, as people in China are less willing to provide their financial information.

Our sampling strategy allows us to do spatial analysis of migrants with the individual coordinate data. The data is to some extent geographically representative. The spatial cluster analysis concluded that the spatial distribution of vaccination migrants did not randomly distribute in the whole city, and we identified 3 clusters of migrants with low HBV vaccination rate, although two of them were not statistically significant. But we still report them as they are with high relative risks and interesting. The 3 clusters are located in the suburban and the exurban areas of the city. The suburban and the exurban areas are less developed and their houses are cheaper compared with the downtown area. Existing studies show that migrants are mostly living in urban villages, usually in suburban and exurban areas [29,30]. Our data shows that 53.6% of participants are living in downtown areas, while cluster analysis demonstrates that most deprived migrants concentrated in urban villages. On the other hand, the spatial analysis implies strategies for further health intervention. In 2012, the Chinese CDC begun promoting adult HBV immunization to reduce the deaths (300,000) from HBV related liver diseases. The spatial clusters of low vaccination rate can help us target the high priority area for the vaccination promotion.

*Limitations of this study should be acknowledged. First, the grouping of pre- and post-1980 migrants leads to the difference on the age distribution between the two groups*. The distribution around mean age is not drastically different between the two groups. In the pre-1980 cohort, the age range is 16-30, and in the post-1980 cohort, only 11.8% is 45 or above. Nonetheless, the age effect could still confound with the period effect. Second, our work lacks the vaccination place for the vaccinated people. Consequently, we cannot identify the exact source of variation for the spatial pattern of vaccination status.

## Conclusion

In conclusion, the study showed that compared to the older generation of migrants born before 1980, the Post-1980 generation migrants get more health protection, implying that migrants should not be treated as a homogenous group. And the spatial analytical techniques illustrate low HBV immunization clusters are highly linked with pre-1980 generation migrants and other socioeconomic factors, especially the employment condition. Such information might shed light on the differences and needs across migrant subgroups and may be useful for designing more targeted health policies for Chinese migrants.
